# Predicting epileptic seizures using nonnegative matrix factorization

**DOI:** 10.1371/journal.pone.0228025

**Published:** 2020-02-05

**Authors:** Olivera Stojanović, Levin Kuhlmann, Gordon Pipa

**Affiliations:** 1 Department of Neuroinformatics, Institute of Cognitive Science, Osnabrück University, Osnabrück, Germany; 2 Data Science and AI Group, Faculty of Information Technology, Monash University, Clayton, Victoria, Australia; University of British Columbia, CANADA

## Abstract

This paper presents a procedure for the patient-specific prediction of epileptic seizures. To this end, a combination of nonnegative matrix factorization (NMF) and smooth basis functions with robust regression is applied to power spectra of intracranial electroencephalographic (iEEG) signals. The resulting time and frequency components capture the dominant information from power spectra, while removing outliers and noise. This makes it possible to detect structure in preictal states, which is used for classification. Linear support vector machines (SVM) with L1 regularization are used to select and weigh the contributions from different number of not equally informative channels among patients. Due to class imbalance in data, synthetic minority over-sampling technique (SMOTE) is applied. The resulting method yields a computationally and conceptually simple, interpretable model of EEG signals of preictal and interictal states, which shows a good performance for the task of seizure prediction on two datasets (the EPILEPSIAE and on the public Epilepsyecosystem dataset).

## Introduction

The ability to predict epileptic seizures provides an opportunity to intervene in order to attenuate their effects, or if possible prevent them. In this study we focus on EEG manifestations of seizures, which are characterized by sudden hypersynchronization of neurons and last from seconds to minutes. [1] Recently published studies on seizure prediction use a wide variety of approaches, from time series analysis (e.g. phase synchronization [2] or bivariate phase synchrony [3]) and spectral features of EEG signals [4, 5] to physiological models of neural activity (e.g. neural mass models [6]) or circadian models [7]. We focus on spectral measures of EEG signals since they have been successfully used as features for seizure prediction, and are easily interpretable. [4, 8, 9]

In the field of seizure prediction there are certain conceptional, computational and data-related challenges. First, using a large number of features for prediction makes it difficult to interpret their individual contribution. [9] Secondly, the algorithms for seizure prediction in a clinical setting need to be computationally efficient. Due to hardware constraints, this applies to closed-loop EEG devices for seizure prediction and intervention in particular, which have been a recent focus in the field. [8–11] Finally, data encountered in the field of seizure prediction can be high dimensional and heterogeneous (e.g. recorded using many different channels and types of measurements in addition to EEG, like ECG, EOG etc), yet suffer from class imbalance (patients spend more time in interictal than in preictal states) and limited in the number of labeled samples. This is particularly challenging for the design of a patient-specific model.

In this study we address these issues by developing an easy-to-use, computationally efficient method for patient-specific seizure prediction. In order to achieve that, we extract a small set of interpretable features from power spectra that distinguish a baseline (interictal) EEG activity from a state leading up to a seizure (preictal state). Interictal states are regular brain activity between seizures, which can sometimes be interrupted with interictal spiking. [1, 12] Since seizures are characterized by strong synchronization, they are very prominent in power spectra of EEG signals. Although preictal states are not clearly visible in raw EEG signals, multiple studies confirmed the presence of distinct preictal states using spectral [4, 13, 14], as well as information measures. [15–17] For a detailed discussion, see [8] and [9].

Although power spectra capture relevant changes in frequency over time, they can be very noisy and contain outliers. We thus use nonnegative matrix factorization (NMF) [18, 19] to decompose power spectra into dominant time and frequency components, which are later used for seizure prediction.

To mitigate class imbalance, we employ synthetic minority over-sampling technique (SMOTE) [20], together with linear SVM with L1 regularization, to assign weights for contributions from each individual channel and eliminate uninformative channels. A software implementation of the presented method is available online at: https://github.com/ostojanovic/seizure_prediction. The method is applied to a part of the Freiburg EPILEPSIAE dataset [21], and compared to the Epilepsyecosystem dataset [22]. The developed method is computationally inexpensive and produces good results while providing insights into the structure of preictal states.

## Materials and methods

### Data preparation

#### Freiburg EPILEPSIAE dataset

The data consist of heterogeneous EEG recordings of five pre-surgical patients (one female; median age: 29.2) [Table 1] and form a part of the bigger Freiburg EPILEPSIAE database. [21] Recordings are made at the University Medical Center Freiburg, over the course of several days (three to nine), between 2003 and 2009. The sampling frequency varies between 256Hz and 1024Hz. The electrodes that are used in the recordings include intracranial (depth, strip and grid) and surface electrodes, together with special electrodes (e.g. ECG, EMG and EOG), whose number varies between 31 and 122, depending on the diagnosis. In order to investigate preictal states thoroughly, only intracranial EEG recordings are used.

**Table 1:** Detailed information about patients the from EPILEPSIAE database. [21] The number of preictal intervals is the same as the number of seizures.

Patient’s number	age	sex	number of channels	sampling frequency (Hz)	number of preictal intervals	number of interictal intervals
1	34	male	48	256	16	88
2	37	female	26	512	6	44
3	18	male	94	1024	8	80
4	42	male	38	1024	6	110
5	15	male	91	256	14	9

Since the ability to predict a seizure five minutes before its onset can be useful for patients with uncontrolled epilepsy [23], we focus on five minute intervals of preictal and interictal states. In the case of a preictal state, an interval of five minutes leading up to a seizure, with a 30 seconds seizure horizon is extracted. Seizure onsets are hand-labeled at the University Medical Center Freiburg. Since preictal states directly precede seizures, seizure prediction can be realized by classification between preictal and interictal states.

In the case of an interictal state five minutes intervals are extracted, which are at least 11 minutes before or after any other seizure. We refer to these intervals of extracted signals as individual measurement periods. The data are filtered with the Parks-McClellan optimal equiripple finite impulse response filter to remove 50Hz line noise.

The dataset is separated into training (70%) and validation set (30%) during a 100-fold cross-validation procedure.

#### Epilepsyecosystem dataset

The dataset consists of intracranial EEG recordings of three patients (all females; median age: 50). [Table 2] Recordings are made at the St Vincent’s Hospital in Melbourne, Australia as a part of the world-first clinical trial of the implantable NeuroVista Seizure Advisory System. [24] In total, 16 electrodes are used for each patient and sampling frequency is 400Hz. The dataset consists of the public and the private (benchmark) set. Since labels of preictal and interictal states are known only for the public set, it is used for developing a model, while the benchmark set is used in the final stage for comparison with other algorithms for seizure prediction. [22]

**Table 2:** Detailed information about the Epilesyecosystem dataset (after excluding corrupted files). [22] The number of preictal intervals is the same as the number of seizures in the public dataset, while for files in the benchmark dataset labels are not publicly known.

Patient’s number	age	sex	number of preictal intervals	number of interictal intervals	number of files (benchmark set)	percentage of excluded files
1	22	female	225	500	162	14.9%
2	51	female	216	1688	941	7%
3	50	female	251	1896	679	1%

Preictal intervals are ten minute segments which are cut out of recordings covering one hour prior to seizure with a five minute seizure horizon. (i.e. from 1:05 to 0:05 before seizure onset). Interictal intervals are also ten minute segments cut out from one hour of recording, which is at least four hours away from any seizure. Some of the files contain data dropouts which happen when the intracranial brain implant temporarily fails to record data. This manifests in zero values of iEEG across all channels at a given time sample. All files that contain more than 50% of data dropouts are excluded from the further analysis. For files that contain less than 50% of data dropouts, the corrupt data are deleted and the rest of the signal is concatenated. The data are filtered with the Butterworth infinite impulse response filter to remove 50Hz line noise.

The public dataset is separated into training (70%) and validation set (30%) during a 100-fold cross-validation procedure.

### Deriving time and frequency components

**Fig 1:**
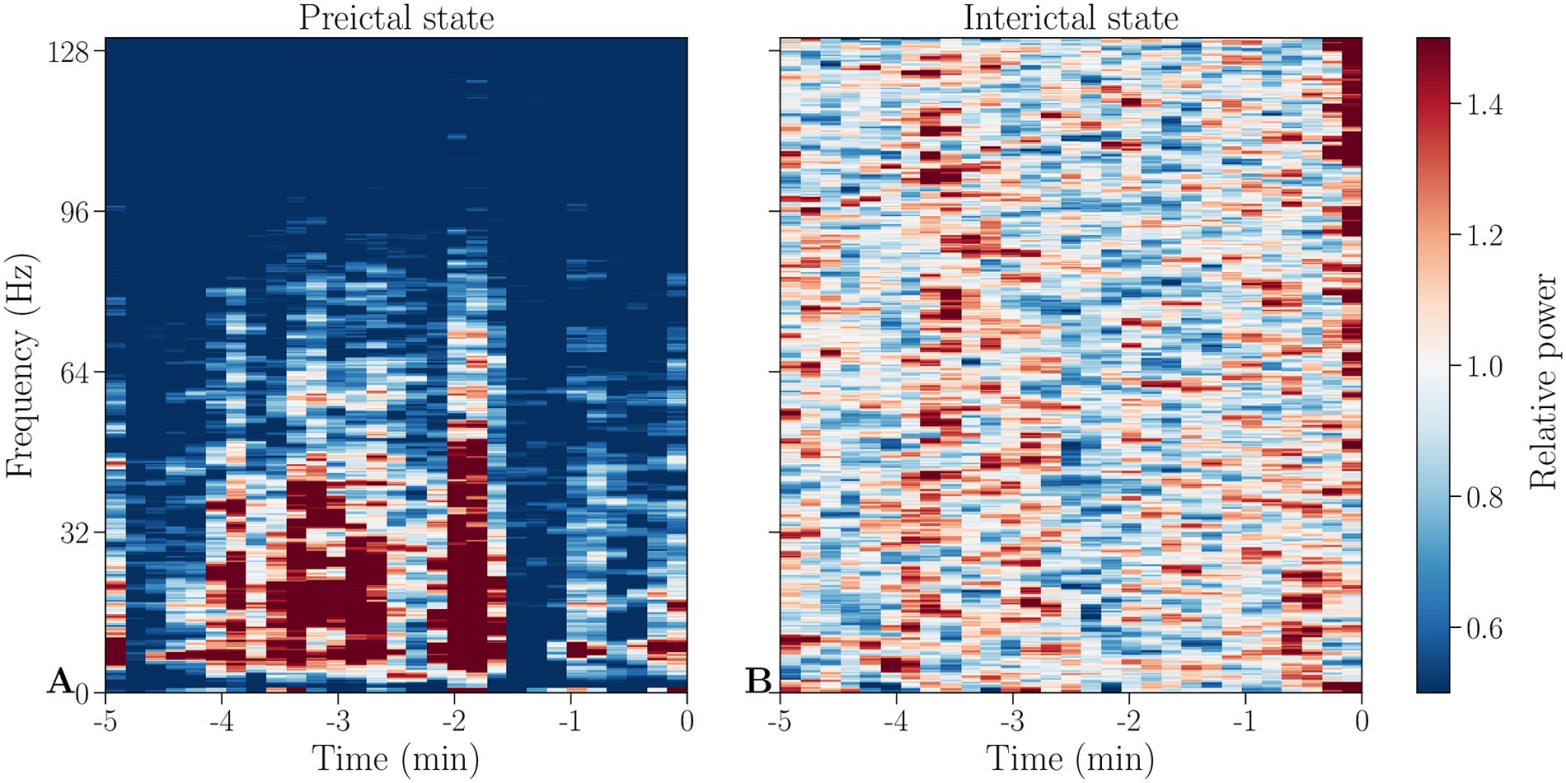
Example spectrograms of preictal and interictal states. Baseline corrected spectrograms of a preictal (**A**) and an interictal (**B**) individual measurement period of channel HR1 from patient 1. This channel and individual measurement period will be used throughout the paper for illustrative purposes, if not stated otherwise.

To identify stereotypical behavior between and ahead of seizures, spectrograms of each channel [Fig 1] (for the Freiburg EPILEPSIAE dataset) are obtained using the multitaper method [25] with time windows of 10 seconds (which is calculated by using 50% overlap of a 20 seconds window). For the Epilepsyecosystem dataset, spectrograms of each channel are calculated using the Fast Fourier Transform. To correct for baseline activity across frequencies, relative power is calculated by dividing spectrograms of each channel by the average interictal spectrogram.

Due to the clinical setting and patients’ diagnoses, the sampling frequency varies among different patients from the two datasets. As a result, the highest frequency in the spectrograms varies between 128Hz and 513Hz. However, this difference is unproblematic due to the fact that we develop patient-specific models. After obtaining spectrograms of every individual measurement period for every channel, they are visually inspected, and in the case of anomalies (e.g. electrode detachments, sudden amplitude jumps), excluded from the data.

#### Time-frequency decomposition

To examine changes in power spectra, spectrograms of each channel and each individual measurement period are decomposed into a time and a frequency component using nonnegative matrix factorization. Originally proposed under the name “positive matrix factorization”, it is a variant of factor analysis [18], which is first used on environmental data [26] and later popularized in the application to face recognition under the current name. [19] For both tasks, NMF is successful in learning interpretable parts-based representation (e.g. concentrations of elements, as in [26] or parts of faces, as in [19]) and shown to perform better than independent component analysis, principal component analysis or vector quantization. [27–29] In the field of seizure prediction, NMF has been used to develop a method for automatic localization of epileptic spikes in children with infantile spasms [30] and for automatic detection and localization of interictal discharges. [31]

Nonnegative matrix factorization decomposes a nonnegative matrix *V* into two nonnegative low-rank matrices *W* and *H* [19]:

$$V∼{\tilde{V}}_{n\times m}=W_{n\times r}\times H_{r\times m}$$

$${\tilde{V}}_{ij}=\sum_{a=1}^{r}W_{ia}H_{aj}$$

**Fig 2:**
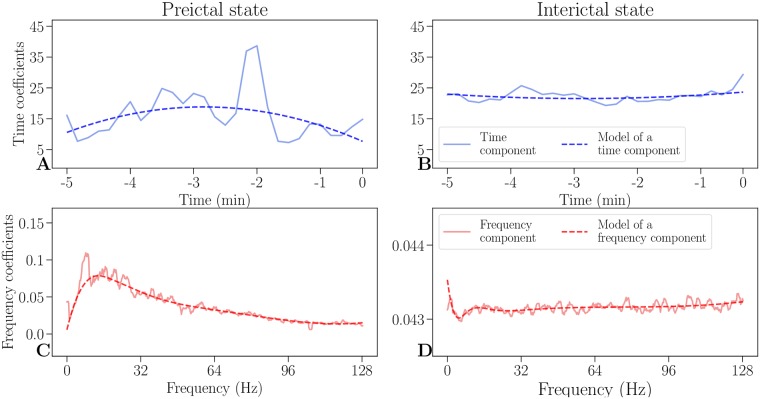
Time and frequency components and its models. An example of decomposed time (solid blue lines) and frequency components (solid red lines) and their respective models (dashed lines) of a preictal state (**A**, **C**), as well as an interictal state (**B**, **D**). In a preictal state, the time component (**A**) increases as a seizure is approaching, while the frequency component (**C**) has an increase in low frequencies. Both interictal components (**B**, **D**) are steady and are an order of magnitude lower than their respective preictal components (**A**, **C**).

The outer product $\tilde{V}=WH$ can be interpreted as a low rank parts-based approximation of the data in *V*. [19] We decide on a factorization of rank *r* = 1 to get the most constrained model with two vectors, one of which represents temporal evolution (time component *H*) and one of which represents distribution of frequencies (frequency component *W*). [Fig 2]

To lessen the influence of outliers and to remove noise in the NMF components, they are modeled with smooth basis functions using robust regression. The time component is modeled by a polynomial of second order, while the frequency component is modeled by nonlinearly logarithmically spaced B-splines of sixth order to consider the frequency resolution which decreases in higher frequencies. [Fig 2] By modeling each component with smooth basis functions, the most relevant information is preserved in both domains, while noise is removed.

**Fig 3:**
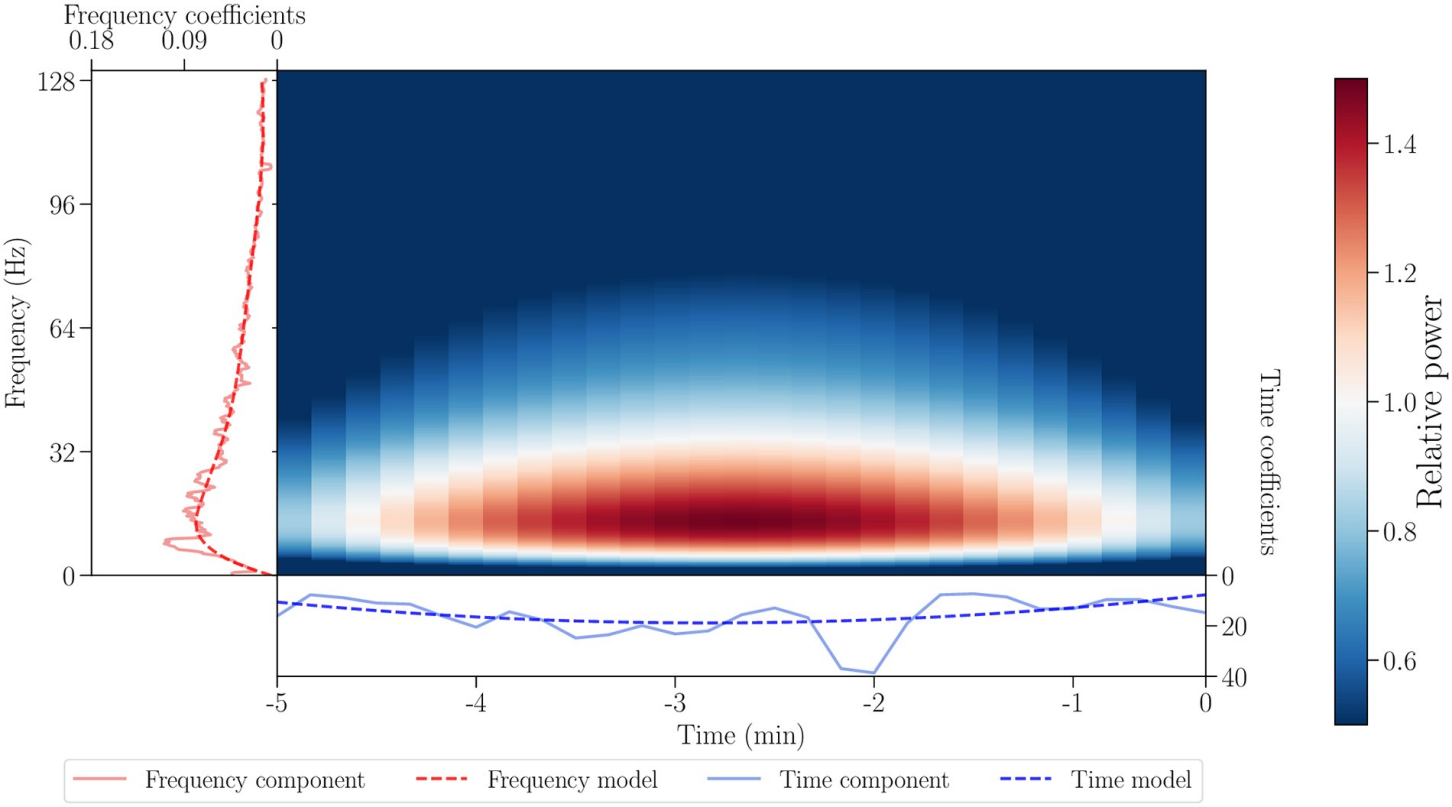
Obtaining a time-frequency model from the respective components. The NMF components are shown with solid red and blue lines for frequency and time, respectively, while their models are shown with dashed lines. The time-frequency model (center) is an outer product of modeled time and frequency components.

By calculating the outer product of modeled NMF components as shown in Fig 3, time-frequency models can be reconstructed. They capture the most important information while leaving out the noise and thus provide simplified intermediate representation of the data, which can be visually compared to the corresponding spectrograms (see S1 Fig in the appendix). The coefficients of the modeled time and frequency components therefore convey relevant information about structure of both states.

### Prediction and performance measures

To classify between preictal and interictal states, linear support vector machines [32] are used. We combine the coefficients of both of the modeled NMF components across all channels into a feature vector. For example, recordings of patient 1 in the EPILEPSIAE dataset contain 48 channels with 12 NMF parameters (9 parameters for the frequency component and 3 parameters for the time component) each, leading to a dimensionality of 48 ⋅ 12 = 576. To account for the risk of overfitting due to the high number of features, L1 regularization is used. L1 regularization shrinks coefficients of less important features to zero by adding the absolute value of magnitude of coefficients as a penalty term to the loss function. [32]

In both datasets, interictal states are more frequent than the preictal ones, which leads to an imbalance of classes (c.f. Tables 1 and 2). To account for this, the SMOTE oversampling technique is used. [20] It creates synthetic samples of the minority class, based on *k* neighboring points of minority samples (in our case *k* = 5). This means that the new synthetic preictal sample is created based on the five closest preictal samples.

To ensure good generalization of the algorithm, 100-fold cross-validation is used on a training set (70%) and a validation set (30%). Average measures (accuracy, sensitivity, specificity, positive and negative predictive values) are reported. Since the classifier should neither miss nor falsely predict a seizure, we report sensitivity sensitivity and specificity, as well as positive and negative predictive values. [33] In the benchmark dataset the area under the curve (AUC) is used for comparison among other algorithms.

Sensitivity is the probability of a positive test result among those having the target condition (i.e. the proportion of correctly classified preictal states), while specificity is the probability of a negative test result among those without the target condition (i.e. the proportion of correctly classified interictal states). [33] The positive predictive value (PPV) is the probability of the target condition, given a positive test result (i.e. the measure of how likely it is that, if the classifier predicts a preictal state, a patient is experiencing it), while the negative predictive value (NPV) is the probability of not having the target condition, given a negative test result (i.e. the measure of how likely it is that, if our classifier does not predict a preictal state, a patient is not experiencing it). [33] Full expressions are given below:

$$\begin{array}{cc} \text{Accuracy} & =\frac{TP+TN}{\text{all}\;\text{samples}}\\ \text{Sensitivity} & =\frac{TP}{TP+FN}\\ \text{Specificity} & =\frac{TN}{TN+FP}\\ \text{PPV} & =\frac{TP}{TP+FP}\\ \text{NPV} & =\frac{TN}{TN+FN} \end{array}$$

where:

**TP** is a number of samples classified as true positive

**TN** is a number of samples classified as true negative

**FP** is a number of samples classified as false positive

**FN** is a number of samples classified as false negative.

## Results and discussion

### Interpretability of the model

Fig 2 shows representative preictal and interictal components (of the EPILEPSIAE dataset), where the modeled NMF components show differences between the states. Model of the frequency component of a preictal state exhibits a peak of high activity in lower frequencies, relative to baseline activity. This is in line with previous findings of a structure below 30Hz (gamma range), which is informative for seizure prediction. [13, 14] These structural differences are also visible in recovered time-frequency models (see S2 and S3 Figs in the appendix).

Average preictal and interictal components of all measurements and electrodes differ in both datasets, as shown in S4 and S5 Figs in the appendix. On average, time components of preictal states in the EPILEPSIAE dataset have higher intensity, and frequency components show increase in lower frequencies (S4 Fig). Equivalent average components in the public Epilepsyecosystem show slightly different behavior. Time components of interictal states have somewhat higher intensity, and frequency components have an increase in lower as well as in higher frequencies. Since labels for the private Epilepsyecosystem dataset are not available, it is not possible to analyze the benchmark dataset in the same way.

**Fig 4:**
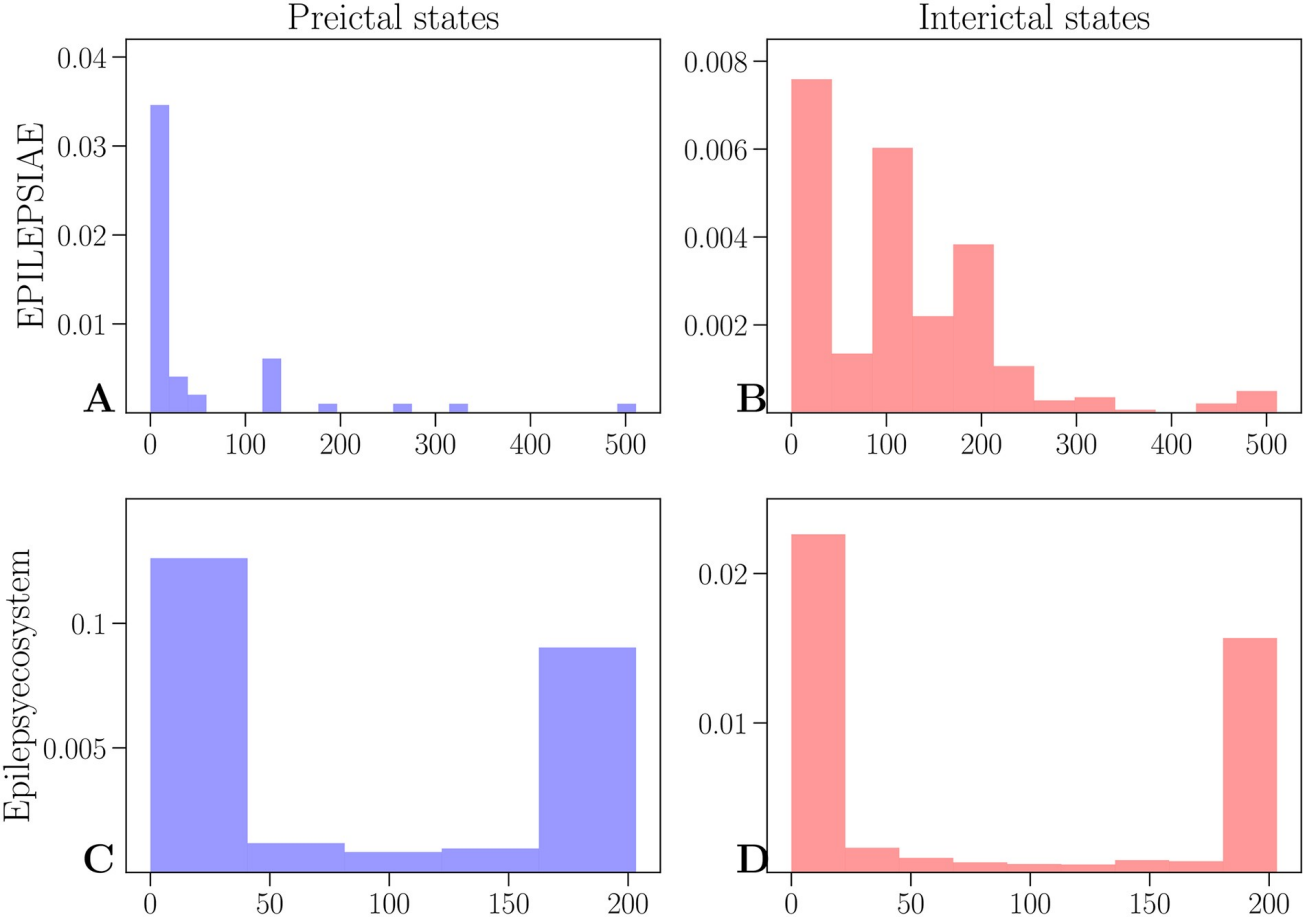
Distribution of maximum of frequency components. Results of the EPILEPSIAE dataset are shown in the upper row for preictal (**A**) and interictal states (**B**). The lower row shows results for the Epilepsyecosystem dataset (**C** for preictal and **D** for interictal states).

Fig 4 shows normalized histograms of maximum values of frequency components of preictal and interictal states for both datasets. In the EPILEPSIAE dataset most preictal components have maximum in lower frequencies, and interictal states have maximum in both lower and higher frequencies (above 100Hz). On the other hand, most maxima of preictal and interictal components in the public Epilepsyecosystem dataset are below 50Hz as well as between 150Hz and 200Hz.

This difference in components between datasets can exist due to various reasons. The part of the EPILEPSIAE dataset used here might have too few measurements from an each patient. The Epilepsyecosystem dataset has more measurements, but it still contains data for only three patients. For a better assessment more data from different patients should be analyzed. In addition, it should be noted that the part of the EPILEPSIAE dataset used here contains data of pre-surgical patients and seizures recorded in this setting might not always be representative of typical epileptic seizures. As it is shown in [34], features of intracranial EEG signals show high variability after implantation of electrodes and spatial variability of lower frequency power bands across channels decreases over time. On the other hand, the Epilepsyecosystem dataset contains recordings from the world-first clinical trial of the human-implanted NeuroVista seizure advisory system [24], which might also be more distinguished than other clinical trials. Lastly, in the EPILEPSIAE dataset the 11-minutes buffer for interictal periods is used, which might be too short. The study in [35] reveals existence of “pre-cursors” to seizures (energy bursts in iEEG signals), which suggests that epileptic seizures might start hours in advance (also shown in [24]). Considering all of this, the best assessment of differences in preictal and interictal states would be in a closed-loop seizure prediction setting in real-time, for which the proposed method would, with appropriate adjustments (e.g. calculating spectrograms of consecutive time windows instead of short segments) be suitable.

### Predictive performance

**Fig 5:**
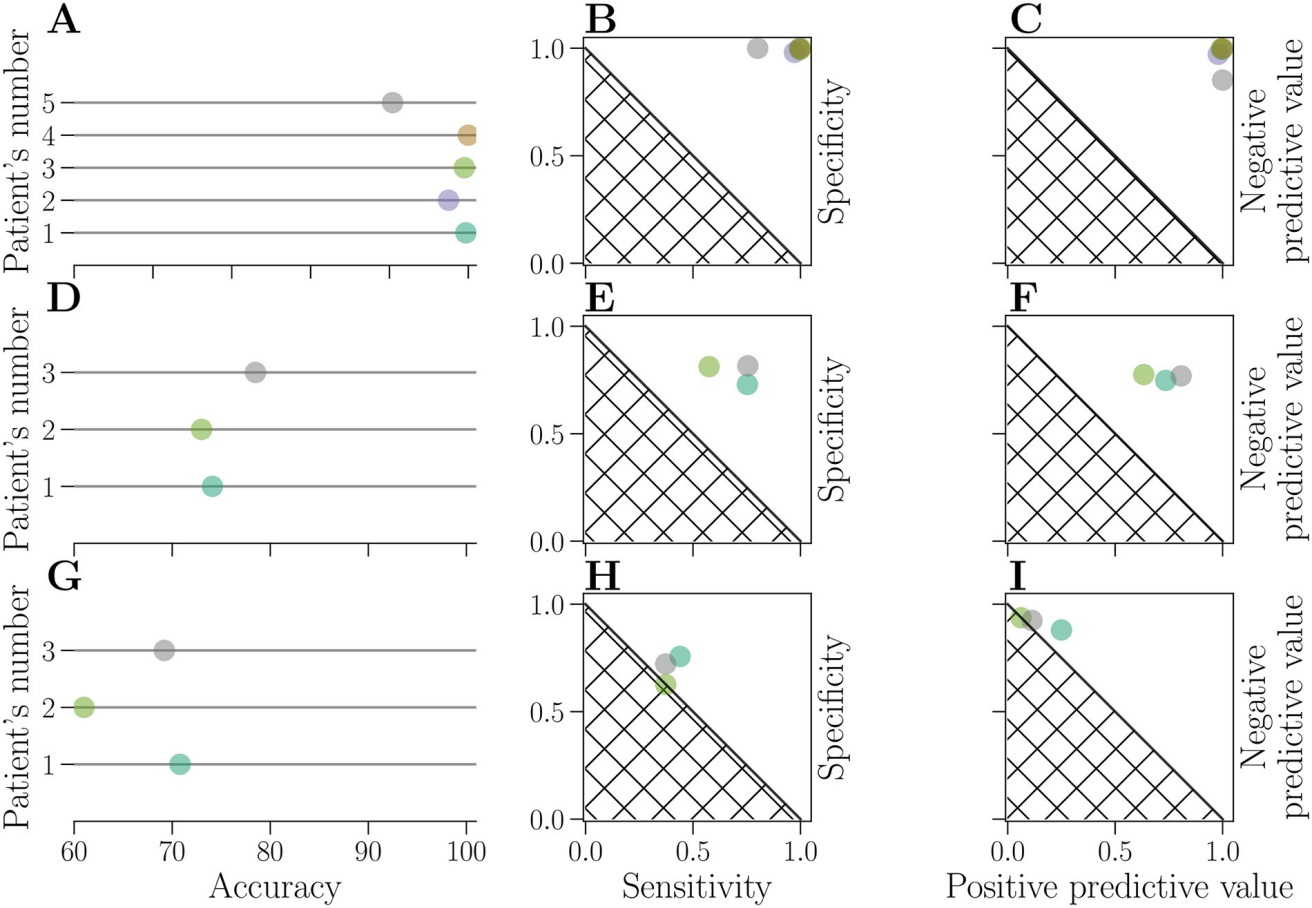
Evaluation of prediction performance. Results on the EPILEPSIAE dataset are shown in the upper row(**A-C**). Results on the public Epilepsyecosystem are shown in the middle row (**D-F**) and the results on the private Epilepsyecosystem dataset (benchmark) are shown in the lower row (**G-I**). Performance of each patient is represented by a circle, for accuracy (**A**, **D**, **G**), specificity-sensitivity plot (**B**, **E**, **H**) and negative and positive predictive value (**C**, **F**, **I**). Identical colors are used to represent each patient across all nine subplots. The hatched area represents results attainable by a random classifier.

On the EPILEPSIAE dataset, similar accuracy is achieved for all patients (above 90%). The lowest performance is for the patient 5 (90.4%) and the highest for the patient 4 (100%), as shown in Fig 5 and Table 3. Sensitivity is between 0.8 and 1, while specificity ranges from 0.98 to 1, as can be seen in Fig 5. A combination of high values of sensitivity and specificity is achieved for all patients. Similarly, positive predictive values are between 0.98 and 1, while negative predictive values are between 0.85 and 1 (c.f. Fig 5 and Table 3).

**Table 3:** Performance measures for all patients from the EPILEPSIAE dataset (upper section), from the Epilepsyecosystem public dataset (middle section) and Epilepsyecosystem benchmark dataset (lower section).

Patient’s number	accuracy (%)	sensitivity	specificity	positive predictive value	negative predictive value
1	99.7	0.99	1	1	0.99
2	97.5	0.97	0.98	0.98	0.97
3	99.5	1	0.99	0.99	1
4	100	1	1	1	1
5	90.4	0.8	1	1	0.85
1	74.1	0.75	0.73	0.73	0.75
2	73	0.57	0.81	0.63	0.77
3	78.5	0.75	0.82	0.81	0.77
1	71	0.44	0.76	0.25	0.88
2	61	0.37	0.63	0.06	0.94
3	69.2	0.37	0.72	0.11	0.92

Predictions on the public Epilepsyecosystem dataset are lower than on the EPILEPSIAE dataset (around 70% for all patients; c.f. Fig 5 and Table 3). The lowest performance is for the patient 1 (74.1%) and the highest for the patient 3 (78.5%). Sensitivity, specificity, positive and negative predictive values for all patients are still higher than attainable results by a random classifier, but still considerably lower than on the EPILEPSIAE dataset, which can be seen in Fig 5. Sensitivity is between 0.57 and 0.75, while specificity ranges from 0.73 to 0.82. Positive predictive values are between 0.63 and 0.81, and negative predictive values are between 0.75 and 0.77.

On the benchmark dataset, the highest achieved accuracy is for the patient 1 (71%), and the lowest for the patient 2 (61%). However, other performance measures drop significantly (sensitivity and positive predictive value are below 0.5). This drop in performance happens with most of other algorithms that are evaluated on the Epilepsyecosystem dataset [22], but the difference is not always as big. There might be various reasons for this. In general, it is the harder task to train a model on one dataset, and then evaluated it on the unseen set. Furthermore, the class imbalance between the sets might differ, which would explain the big difference between sensitivity and positive predictive value. It is also possible that SMOTE algorithm learns noise when oversampling the minority class in the public dataset. Finally, patients who have a higher seizure frequency (i.e. seizures per day) seem to have worse seizure prediction performance based on the original clinical trial. [24]

As mentioned in the *Prediction and performance measures*, the AUC is used for comparison with other algorithms on the benchmark set. The average reported AUC is 0.57 (0.62 for the patient 1, 0.52 for the patient 2 and 0.58 for the patient 3), which places the proposed algorithm on the 65th place (out of current 102 evaluated algorithms). For comparison, the algorithm with the best performance on the benchmark dataset (which is the combination of extreme gradient boosting, k-nearest neighbours, generalized linear model and linear SVM) has AUC of 0.8. [22]

The reasons for the overall lower performance on both Epilepsyecosystem datasets can lie in the fact that there are more seizures and more data per patient, making prediction possibly more challenging by potentially adding more variability to the data. It should also be noted that the data of three patients from the Epilepsyecosystem dataset correspond the ones whose seizures are the most difficult to predict [24].

## Conclusion

Since patients with uncontrolled epilepsy prefer to be advised a few minutes before a seizure onset [23], we decided to use intervals of five minutes, extracted from longer recordings of the EPILEPSIAE dataset. However, this method is easily extensible to longer periods of time, since the length of intervals has no effect on dimensionality of modeled time components, which is shown by comparing the proposed method on the Epilepsyecosystem dataset.

Data from additional patients as well as more data from the same patient could, if available, lead to a better generalization of the model. This however is a challenge for patient-specific models in general, where data from a single patient should suffice, and a large number of labeled training examples is not available.

Overall, this study demonstrates the use of nonnegative matrix factorization of power spectra for a seizure prediction task. The proposed model is conceptually simple, interpretable and has shown good accuracy on two representative datasets and lower performance on the benchmark set where improvements in the direction of coping with class imbalance should be made. A similar approach could be used for similar tasks such as detection of sleep stages in EEG or the detection of irregularities in ECG.

## Supporting information

S1 Fig Time-frequency models and corresponding spectrograms of preictal and interictal states. An outer product of modeled time and frequency components (**A**, **C**) and corresponding spectrograms (**B**, **D**). A preictal state is shown in the upper row (**A-B**) and an interictal state is shown in the bottom row (**C-D**).
(PDF)

S2 Fig Models of preictal states. Models shown here are for different channels (**A-I**) from the same individual measurement period for patient 1.
(PDF)

S3 Fig Models of interictal states. Models shown here are for different channels (**A-I**) from the same individual measurement period for patient 1.
(PDF)

S4 Fig Average models of time and frequency components of all channels and all measurements for preictal and interictal states of the EPILEPSIAE dataset. Models of time components are shown in the upper row (**A-E**), and models of frequency components are shown in the bottom row (**G-K**). Preictal states are indicated with a dashed line and interictal states are indicated with a line marked with + in blue for models of time and red for models of frequency components, respectively.
(PDF)

S5 Fig Average models of time and frequency components of all channels and all measurements for preictal and interictal states of Epilepsyecosystem dataset. Models of time components are shown in the upper row (**A-C**), and models of frequency components are shown in the bottom row (**D-F**). Preictal states are indicated with a dashed line and interictal states are indicated with a line marked with + in blue for models of time and red for models of frequency components, respectively.
(PDF)
