# Supplementary material for: Predicting epileptic seizures using nonnegative matrix factorization
Source: PLoS One. 2020 Feb 5;15(2):e0228025. doi: 10.1371/journal.pone.0228025 (PMC7001919; doi:10.1371/journal.pone.0228025)
Supplement: S1 Fig — An outer product of modeled time and frequency components (A, C) and corresponding spectrograms (B, D). A preictal state is shown in the upper row (A-B) and an interictal state is shown in the bottom row (C-D). (PDF) [file pone.0228025.s001.pdf]

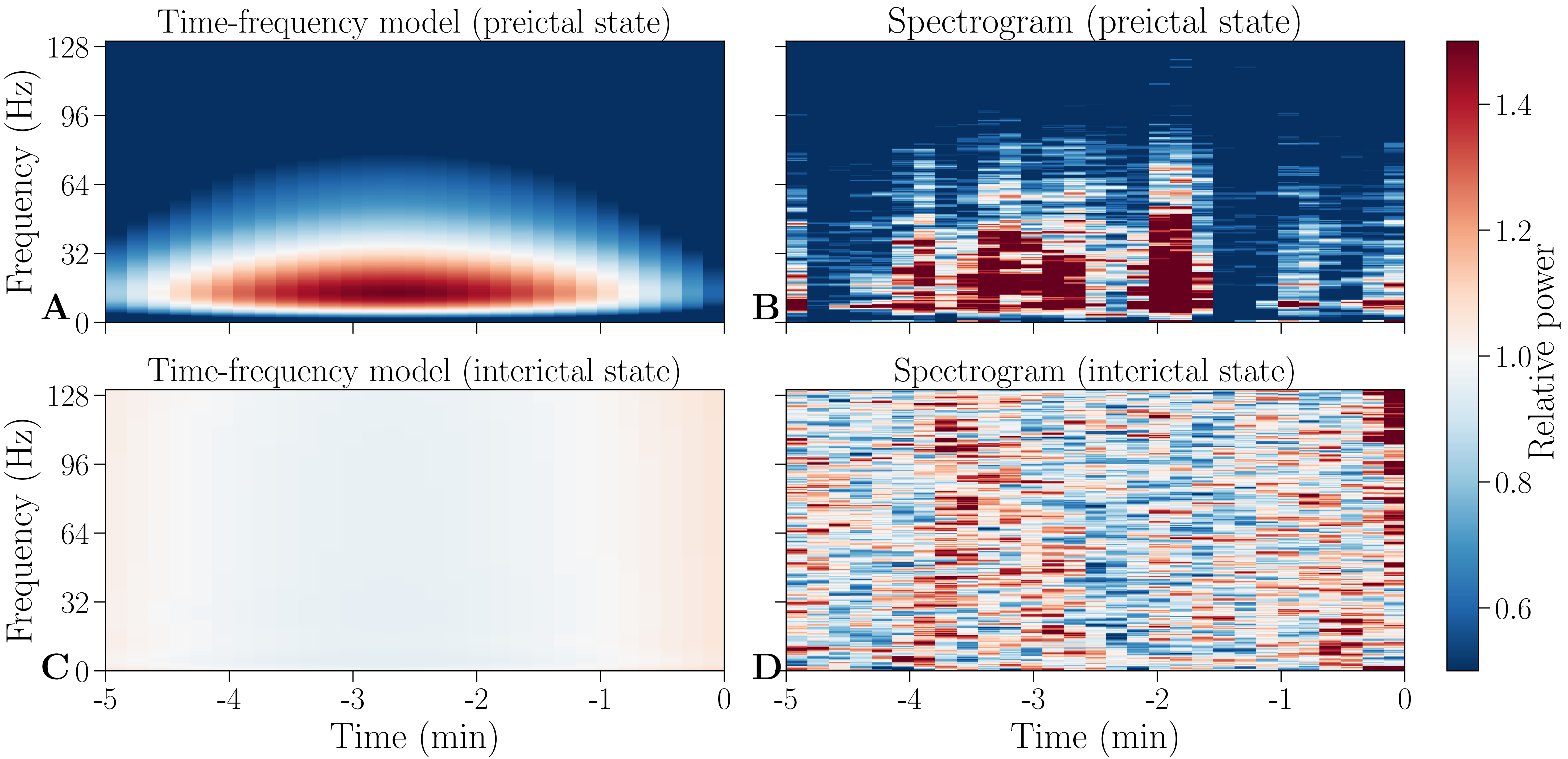

Time-frequency model (preictal state)
Spectrogram (preictal state)
Time-frequency model (interictal state)
Spectrogram (interictal state)
Frequency (Hz)
128
96
64
32
0
Time (min)
-5
-4
-3
-2
-1
0
A
B
C
D
Relative power
1.4
1.2
1.0
0.8
0.6
