# Supplementary figures and images for: Predicting epileptic seizures using nonnegative matrix factorization
Source: PLoS One. 2020 Feb 5;15(2):e0228025. doi: 10.1371/journal.pone.0228025 (PMC7001919; doi:10.1371/journal.pone.0228025)

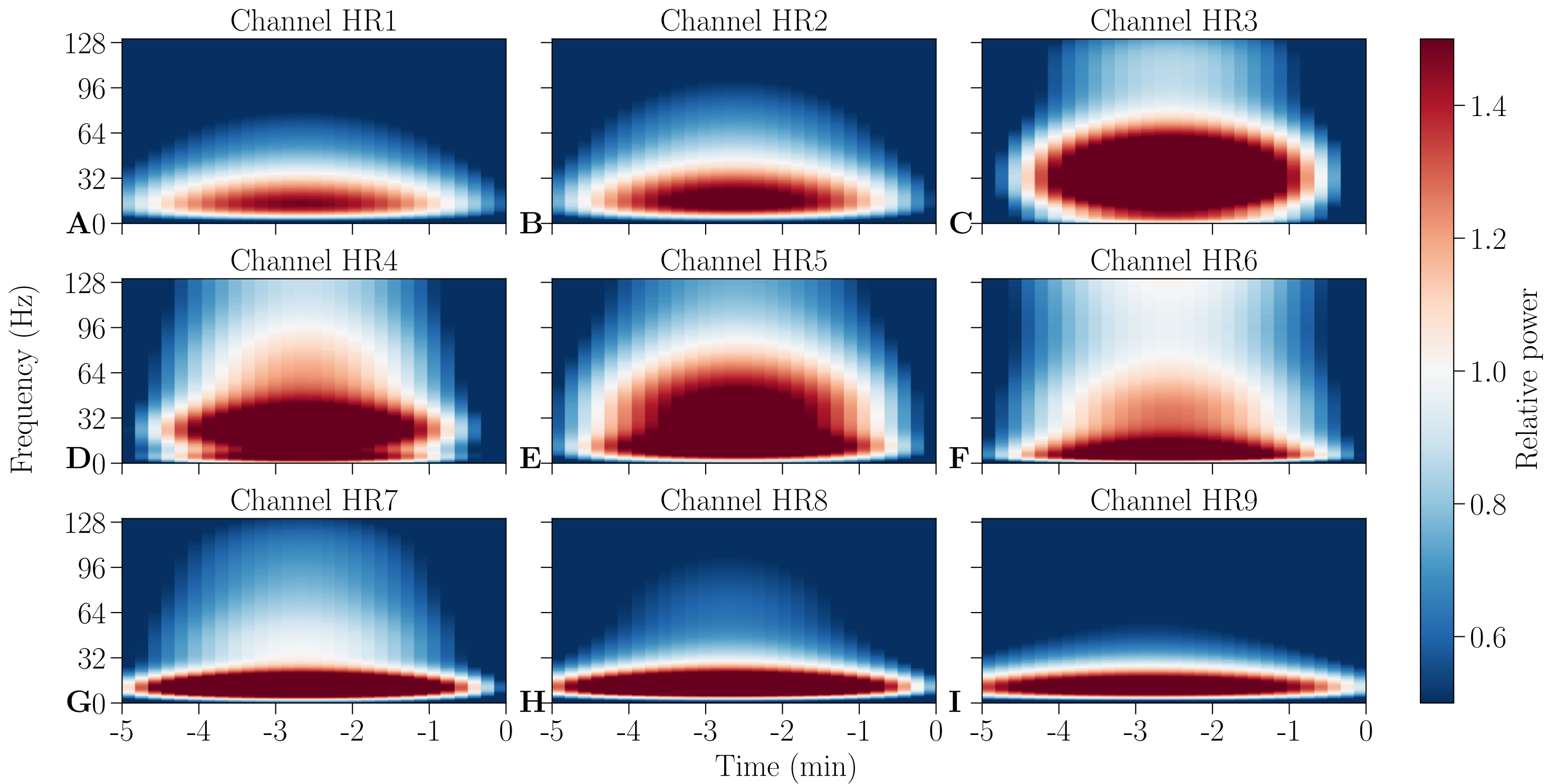

Supplement: S2 Fig — Models shown here are for different channels (A-I) from the same individual measurement period for patient 1. (PDF) [file pone.0228025.s002.pdf]

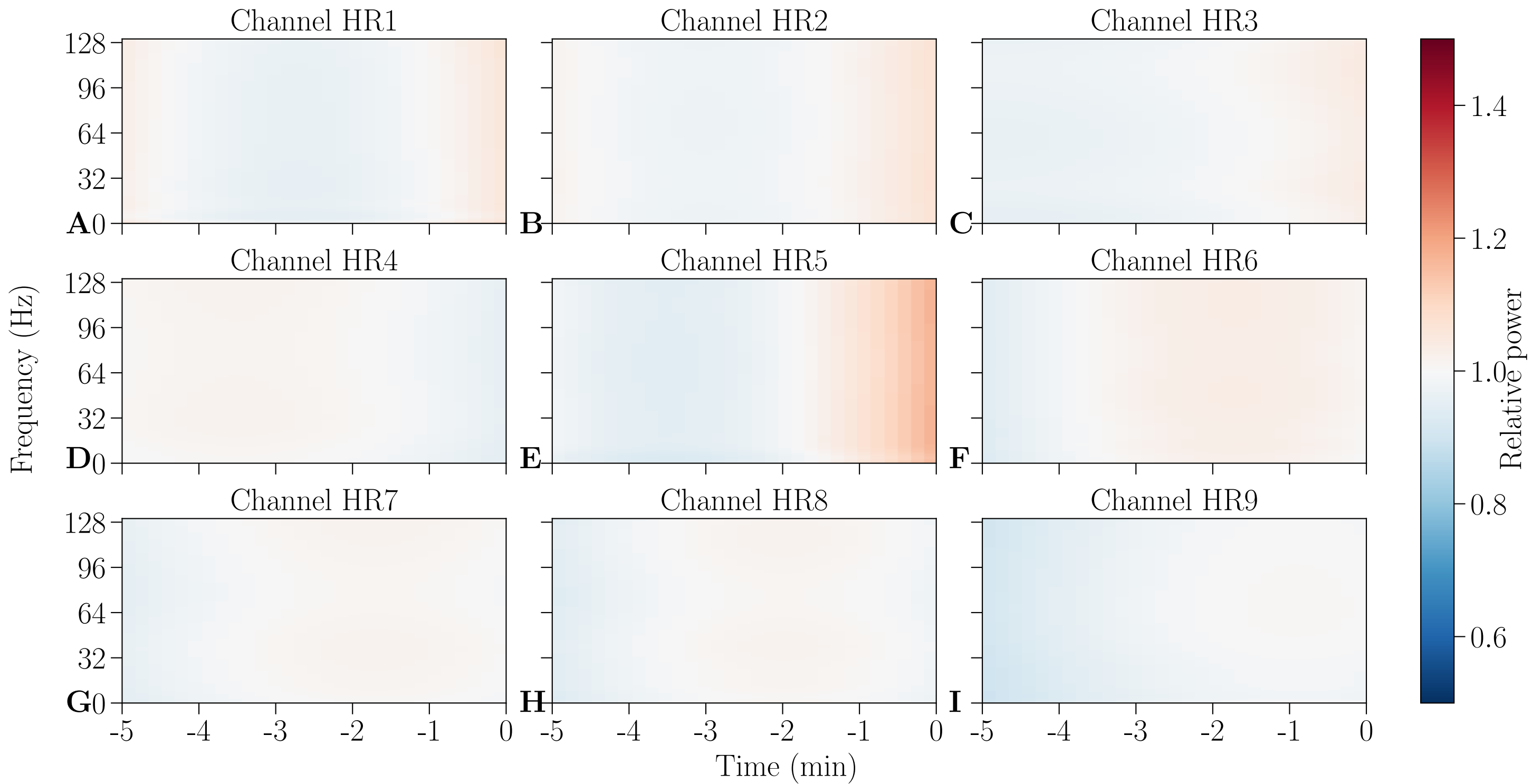

Supplement: S3 Fig — Models shown here are for different channels (A-I) from the same individual measurement period for patient 1. (PDF) [file pone.0228025.s003.pdf]

Patient 1

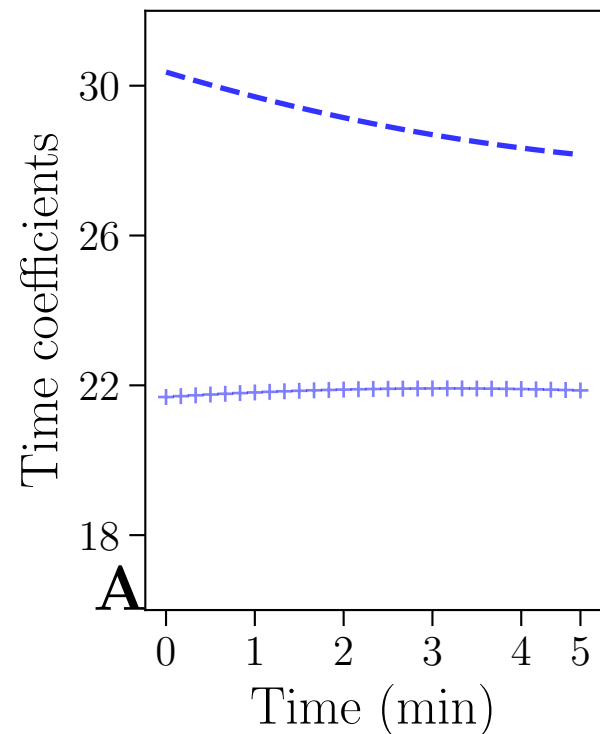

Patient 2

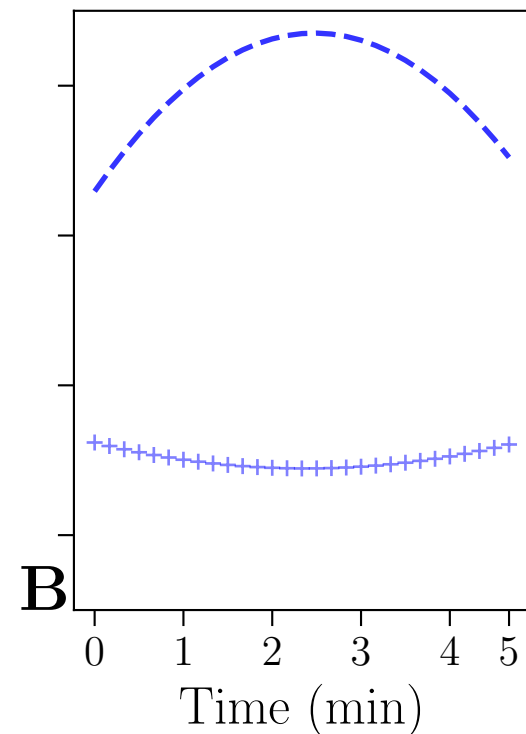

Patient 3

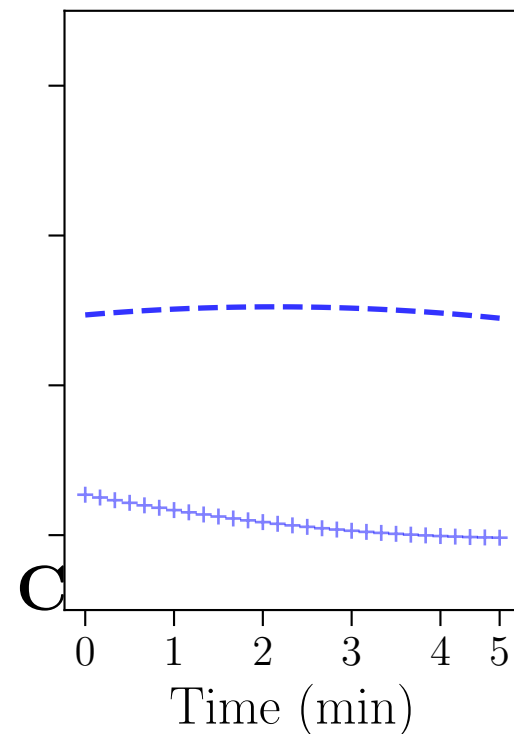

Patient 4

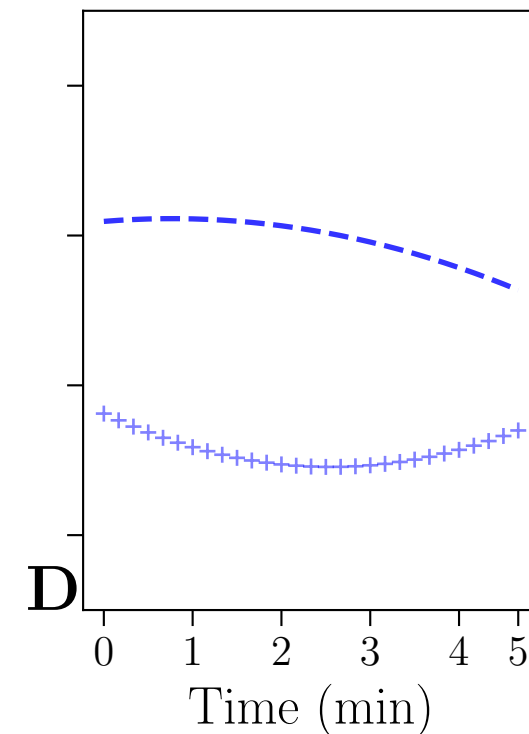

Patient 5

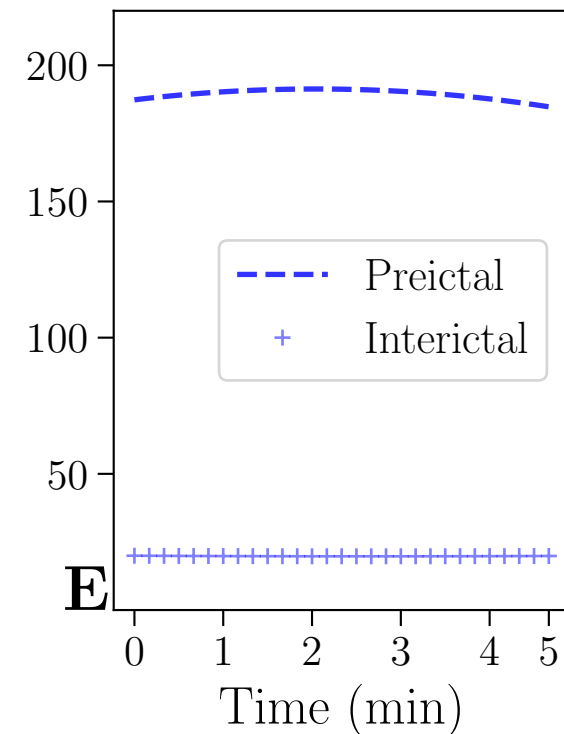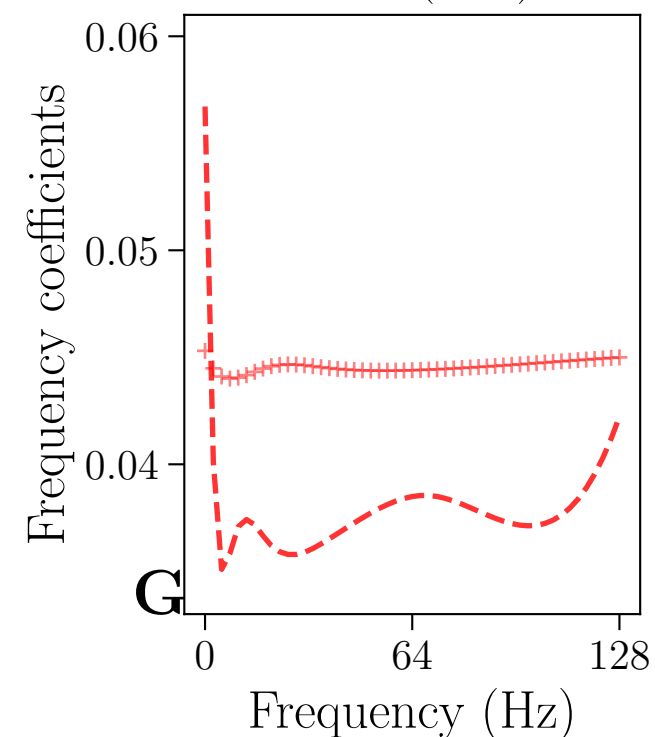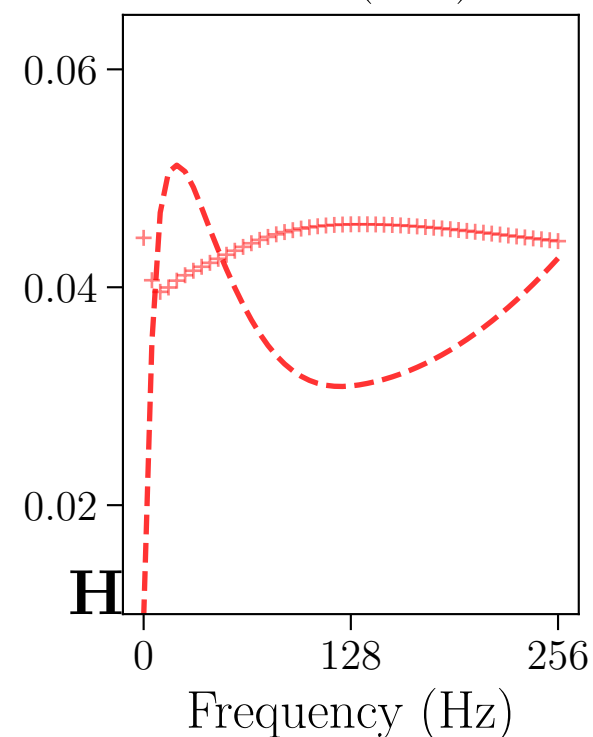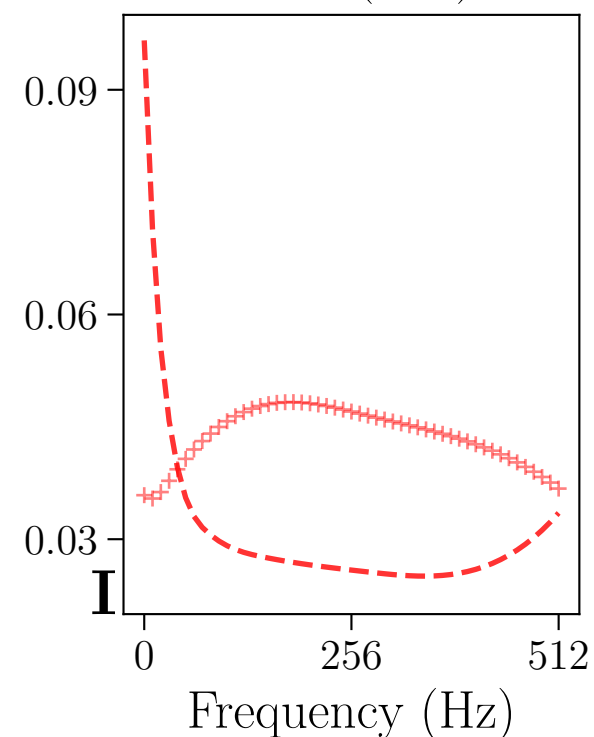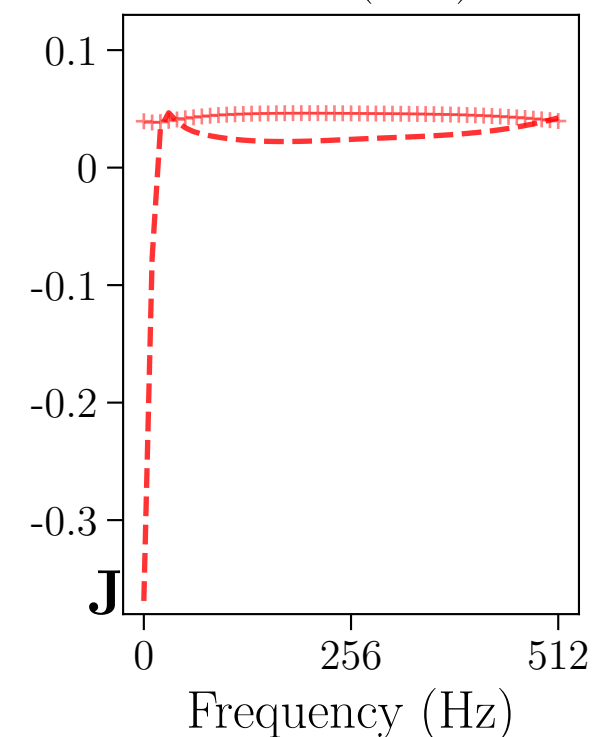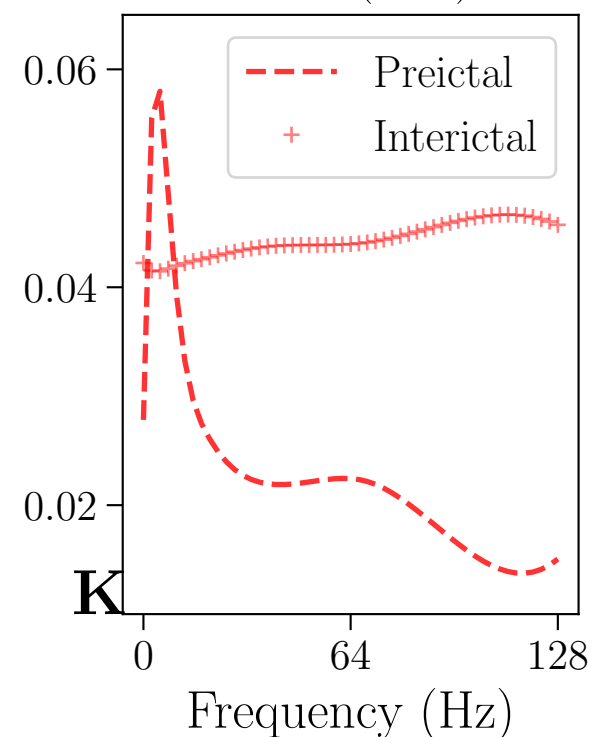

Supplement: S4 Fig — Models of time components are shown in the upper row (A-E), and models of frequency components are shown in the bottom row (G-K). Preictal states are indicated with a dashed line and interictal states are indicated with a line marked with + in blue for models of time and red for models of frequency components, respectively. (PDF) [file pone.0228025.s004.pdf]

Patient 1

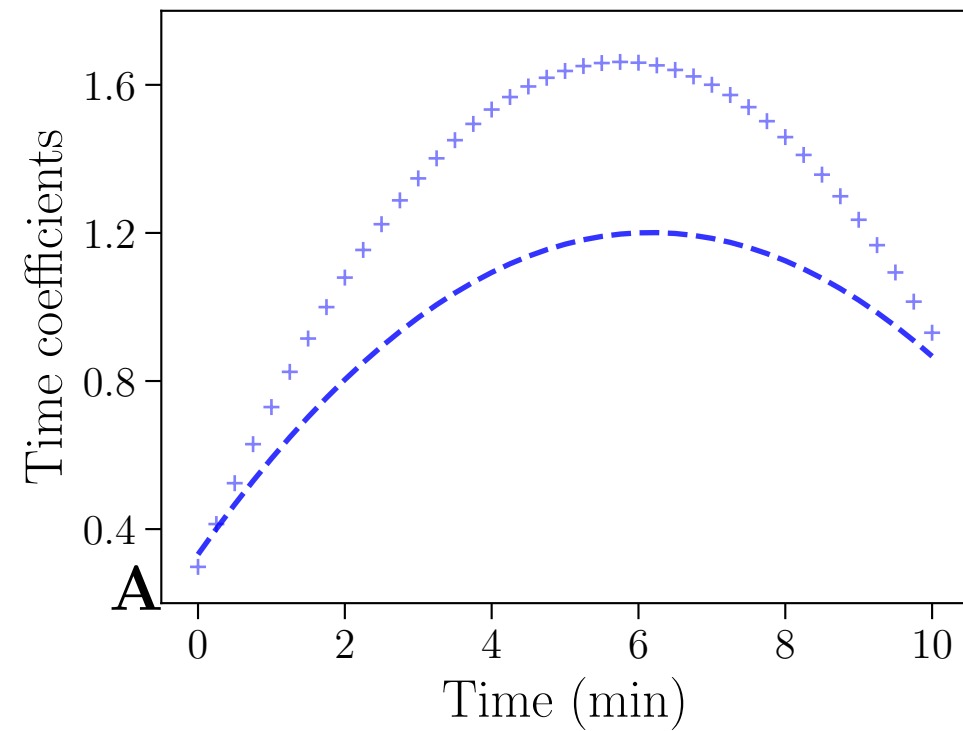

Patient 2

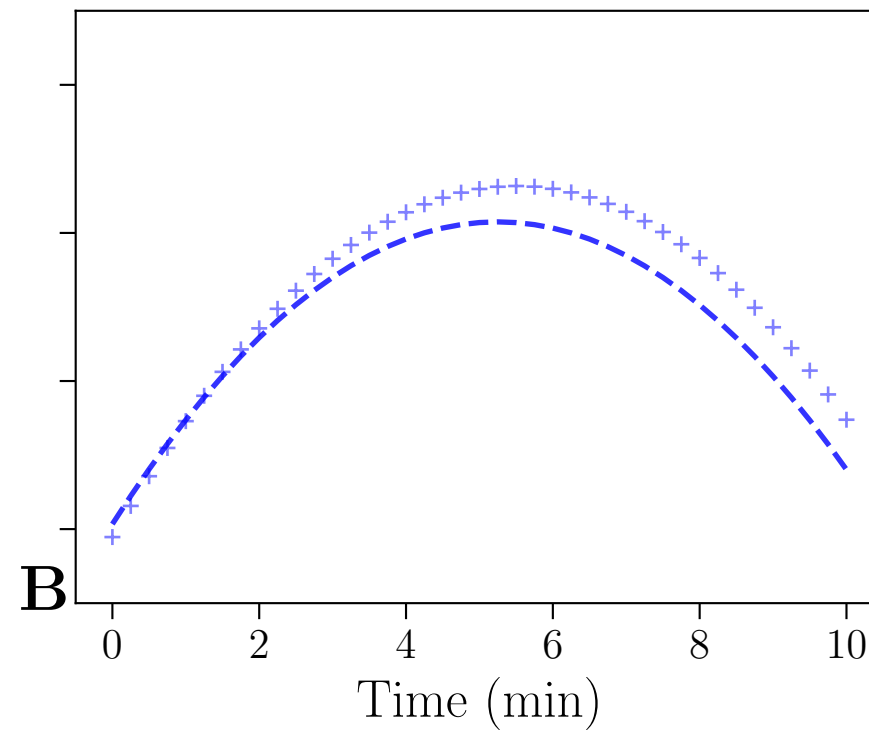

Patient 3

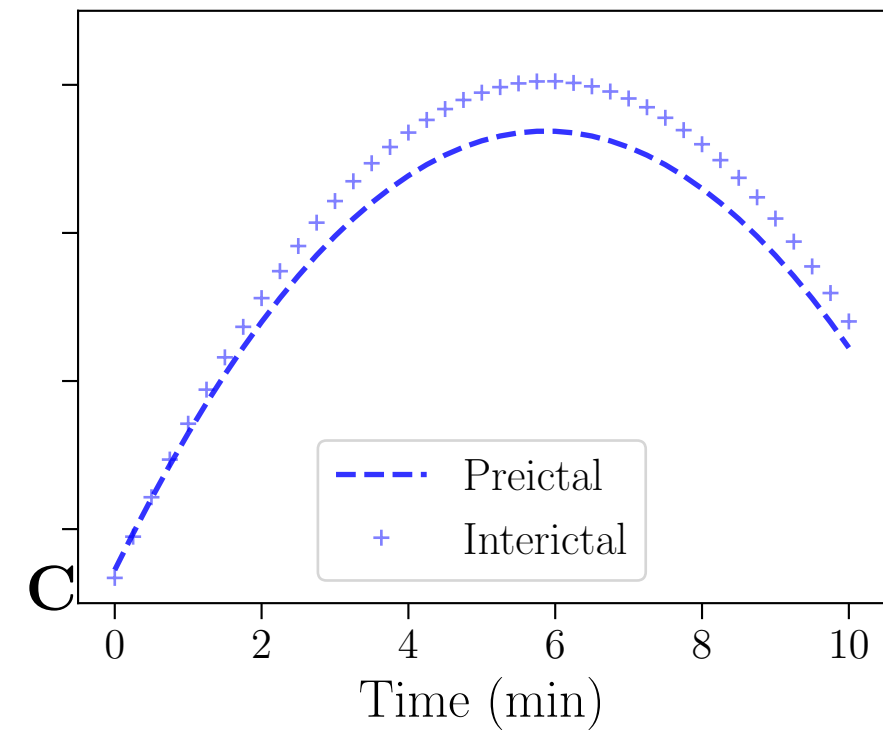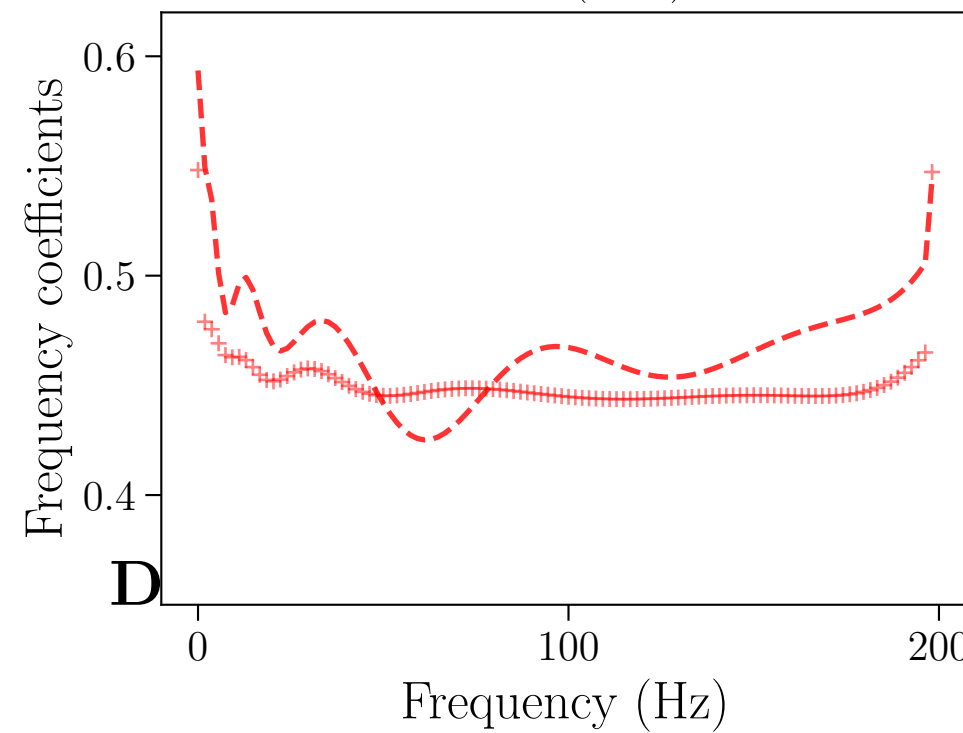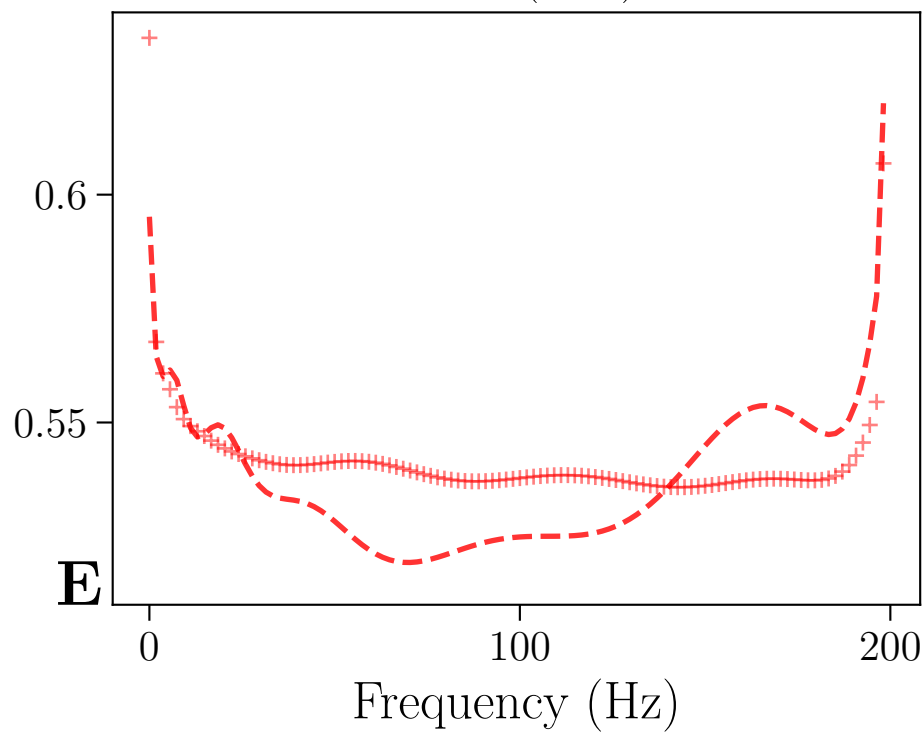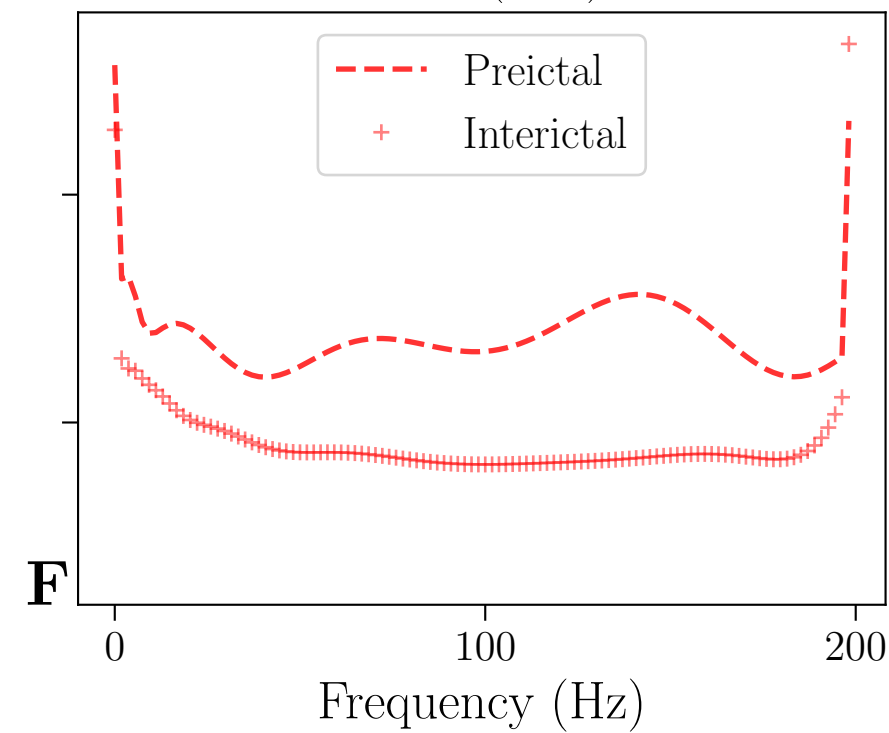

Supplement: S5 Fig — Models of time components are shown in the upper row (A-C), and models of frequency components are shown in the bottom row (D-F). Preictal states are indicated with a dashed line and interictal states are indicated with a line marked with + in blue for models of time and red for models of frequency components, respectively. (PDF) [file pone.0228025.s005.pdf]
